# The Role of PPAR**γ** in the Transcriptional Control by Agonists and Antagonists

**DOI:** 10.1155/2012/362361

**Published:** 2012-05-29

**Authors:** Tamotsu Tsukahara

**Affiliations:** Department of Integrative Physiology and Bio-System Control, Shinshu University School of Medicine, 3-1-1 Asahi, Matsumoto, Nagano 390-8621, Japan

## Abstract

In recent years, peroxisome proliferator-activated receptor gamma (PPAR*γ*) has been reported to be a target for the treatment of type II diabetes. Furthermore, it has received attention for its therapeutic potential in many other human diseases, including atherosclerosis, obesity, and cancers. Recent studies have provided evidence that the endogenously produced PPAR*γ* antagonist, 2,3-cyclic phosphatidic acid (cPA), which is similar in structure to lysophosphatidic acid (LPA), inhibits cancer cell invasion and metastasis *in vitro* and *in vivo*. We recently observed that cPA negatively regulates PPAR*γ* function by stabilizing the binding of the corepressor protein, silencing mediator of retinoic acid and thyroid hormone receptor. We also showed that cPA prevents neointima formation, adipocyte differentiation, lipid accumulation, and upregulation of PPAR*γ* target gene transcription. We then analyzed the molecular mechanism of cPA's action on PPAR*γ*. In this paper, we summarize the current knowledge on the mechanism of PPAR*γ*-mediated transcriptional activity and transcriptional repression in response to novel lipid-derived ligands, such as cPA.

## 1. Introduction

 Nuclear receptors (NRs) bind to small lipophilic molecules, such as steroids [1] thyroid hormones and active forms of retinoids [2]. Peroxisome proliferator-activated receptors (PPARs) were originally cloned as orphan receptors in 1990 [1, 3]. There are 48 members encoded in the human genome [4]. Subsequently, several clinical studies were performed on clofibrates as ligands for PPAR*α* [5, 6]. PPAR*α* is highly expressed in the liver and is considered the key player in the hepatic fasting response [7, 8]. Clofibrates are a pharmaceutical tool for reducing triglyceride levels and increasing high-density lipoprotein (HDL) cholesterol [9]. Other closely related receptors encoded by different genes were subsequently cloned and named PPAR*δ* [10] and PPAR*γ* [11].

 PPAR*γ* is a member of the nuclear receptor gene family that plays a central role in the regulation of glucose and lipid homeostasis. Activation of PPAR*γ* by thiazolidinediones (TZDs) leads to altered metabolism in adipose tissue, skeletal muscle cells, and liver, resulting in insulin sensitization [12]. PPAR*γ* agonists also promote adipocytic differentiation of 3T3-L1 cells and stimulate the uptake of low-density lipoprotein (LDL) by macrophages, leading to foam cell formation in the arterial wall [13, 14]. There is considerable evidence supporting a deleterious role for oxidized phospholipids and fatty acids as important signaling molecules in the context of atherosclerotic lesions [15]. Rother et al. reported that lysophosphatidic acid (LPA) G protein-coupled receptor (GPCR) antagonists abolish platelet aggregation elicited by mild oxidation of LDL (mox-LDL), indicating that LPA plays an essential role in the thrombogenic effects of mox-LDL [16]. When applied topically to the carotid artery wall in rodents, LPA and the TZD drug rosiglitazone induced PPAR*γ*-mediated intimal thickening [13]. Although their functional roles in the PPAR*γ* transcriptional pathway are not well defined, we recently found that production of cyclic phosphatidic acid (cPA), a simple phospholipid, inhibits transcription of PPAR*γ* target genes that normally drive adipocytic differentiation, lipid accumulation in macrophages, and arterial wall remodeling [14]. We also investigated the structure-activity relationship of activation by naturally occurring lysophospholipids. We found that cPA inhibits PPAR*γ* [14, 17] with high specificity through stabilizing its interaction with the corepressor, silencing mediator of retinoic acid and thyroid hormone receptor (SMRT) [14]. These results suggest that cPA is partly mediated by the PPAR*γ* signaling pathway. In this paper, we focus on recent advances in the understanding of the interaction of PPAR*γ* with lipid-derived ligands, particularly focusing on the regulation of PPAR*γ* in response to the endogenous lysophosphatidic acid analogs LPA, alkyl-LPA, and cPA.

## 2. Mechanism of PPAR*γ*-Mediated Effects

### 2.1. Agonist Regulation of PPAR*γ*

PPAR*γ* is most often implicated in lipid metabolism and insulin sensitivity [18, 19]. There are 2 PPAR*γ* isoforms, PPAR*γ*_1_ and PPAR*γ*_2_. PPAR*γ*_2_ has 30 additional amino acids at the N-terminus in humans [20] and is generated from the same gene by mRNA splicing [21]. While PPAR*γ*_1_ is expressed with a broad tissue distribution, PPAR*γ*_2_ is highly expressed in adipocytes [22], adipose tissue [19], macrophages [23], stomach [24, 25], and colon [26–28]. The role of PPAR*γ* has been extensively studied, and a variety of synthetic and physiological agonists have been identified. Several lines of study have suggested that the binding of different PPAR*γ* ligands can induce a range of distinct PPAR*γ* conformations [29]. PPAR*γ* contains a DNA-binding domain (DBD) that binds to hormone response elements in the promoter of its target genes. Upon agonist binding, PPAR*γ* forms a heterodimer with retinoid X receptors (RXRs). PPAR*γ* activation induces a conformational change in the ligand-dependent activation domain (AF-2 helix) located in the c-terminal ligand-binding domain (LBD), which allows coactivator recruitment, corepressor release, and formation of the heterodimeric PPAR*γ*-RXR complex. PPAR*γ*-RXR heterodimer binds the peroxisome proliferator response element (PPRE) in the promoter region of the target genes [30, 31]. The PPAR*γ*-LBD is composed of 13 *α*-helices and a small 4-stranded *β*-sheet that forms a ~1440-Å hydrophobic ligand-binding pocket of the nuclear receptor, which binds many different ligands [32]. Together, these findings suggest that these domains are involved not only in ligand recognition but also in protein-protein interactions.

### 2.2. Synthetic and Natural PPAR*γ* Agonists

**Figure 1:**
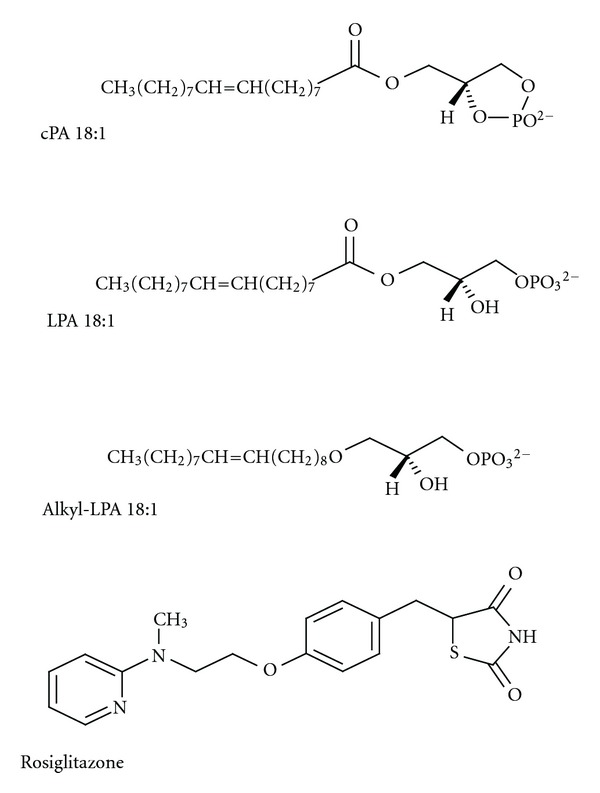
Structural formulas of LPA, alkyl-LPA, cPA,and rosiglitazone. LPA is made up of a glycerol backbone with a hydroxyl group, a phosphate group, and a long-chain saturated or unsaturated fatty acid. Alkyl-LPA is an alkyl-ether analog of LPA. Alkyl-LPA shows a higher potency than LPA at the intracellular LPA receptor PPAR*γ*. cPA is a naturally occurring acyl analog of LPA. cPA is a weak agonist of plasma membrane LPA receptors, whereas cPA is an inhibitor of PPAR*γ*. Rosiglitazone is a thiazolidinedione (TZD) class of antidiabetics and is full agonist of PPAR*γ*.

In the last decade, both synthetic and natural PPAR*γ* agonists have been explored for their biological and physiological functions [33]. Synthetic PPAR*γ* agonists, which include rosiglitazone (Avandia) (Figure 1) [34, 35], troglitazone (Rezulin, withdrawn by the FDA due to causing liver failure) [36, 37], and pioglitazone (Actos; Takeda Pharmaceutical Ltd.) [38, 39], have provided insight into the therapeutic potential of PPAR*γ*. These compounds are specific PPAR*γ* ligands with *K*_*d*_ *s* in the 40–500 nM range [34, 40]. They are effective as insulin-sensitizing agents, reducing insulin resistance and lowering plasma glucose levels in patients with type II diabetes (previously known as noninsulin-dependent diabetes mellitus, NIDDM). Recently, these drugs have also been found to be effective in regulating cell proliferation and differentiation [25]. PPAR*γ* activation by its ligands can induce growth arrest, differentiation, and apoptosis of cancer cells. Similarly, PPAR*γ* heterozygous knockout mice have increased susceptibility to chemical carcinogens [41]. Nevertheless, these reports remain controversial and are not well supported. For instance, low concentrations of PPAR*γ* ligands increase cell proliferation, while high concentrations inhibit cell growth in MDA-MB-231 breast cancer cells [42]. The effective clinical dose of rosiglitazone used in diabetes is 0.11 mg/kg/day [43]. In contrast, the antitumor activity of rosiglitazone in mice requires 100–150 mg/kg/day [43], which is 1,000-fold higher. Therefore, the dosage of PPAR*γ* agonists for cancer therapy must be carefully defined in clinical trials. A recent report suggested that physiological agonists included polyunsaturated acids, such as eicosapentaenoic acid (EPA) [44], linoleic acid [45], and oxidized fatty acid metabolites, cyclopentenone prostaglandin 15-deoxy-Δ^12,14^ (15d-PGJ_2_) [46], 8(S)-hydroxyeicosatetraenoic acid (8(S)-HETE) [47], and the lipoxygenase product, 9-hydroxyoctadecadienoic acid (HODE) [23]. These results were surprising, because these compounds are known to mediate their biological effects through interacting with cell-surface GPCRs, including prostaglandin D_2_ receptors (DP)_1-2_ and G protein-coupled receptor 44 (GPR44), prostaglandin E receptors (EP)_1-4_, prostaglandin F receptor (FP), prostacyclin receptors (IP)_1-2_, and thromboxane receptors (TP). However, in 1995, Forman et al. first reported that the prostaglandin J_2_ derivative, 15d-PGJ_2_, was a natural intracellular agonist of PPAR*γ* as well as a factor of adipocyte determination [46]. 15d-PGJ_2_ is a product of the cyclooxygenase pathway and is the final metabolite of prostaglandin D_2_ (PGD_2_). Some J-series prostaglandins have been found to bind to PPAR*γ* in the low micromolar range [48]. Although 15d-PGJ_2_ was initially identified as a high-affinity endogenous ligand (*K*_*d*_ = 300 nM) [46], the physiological role of 15d-PGJ_2_ remains unclear. In particular, its concentration *in vivo* is much lower than that required for its biological functions [49]. Furthermore, apoptosis induced by 15-PGJ_2_ occurs independently of PPAR*γ* activation and may result from a loss of mitochondrial membrane potential and the formation of reactive oxygen species (ROS) [50, 51].

### 2.3. Lipid-Derived PPAR*γ* Agonists

A number of natural ligands for PPAR*γ* have been identified and include 2 main groups of compounds, fatty acids, and phospholipids. More recently, select phospholipids, such as LPA [52], alkyl-glycerophosphate (alkyl-LPA) [53], hexadecyl azelaoyl phosphatidylcholine (azPC) [54], and nitrolinoleic acid and related metabolites [55], have been identified. LPA (Figure 1) has been reported as a bioactive lipid and is derived from hydrolysis of plasma membrane phospholipids [56, 57]. LPA is already wellestablished as a ligand for specific LPA GPCRs belonging to the endothelial cell differentiation gene family [58] and is formed during mox-LDL [13]. Although exogenous LPA can activate PPAR*γ* [52, 59], the reported *K*_*d*_ of PPAR*γ* with acyl-LPA(18 : 1) is in the high micromolar range, which is at least an order of magnitude higher than its physiological concentration [52]. Examining the specificity of lipid-derived ligands, such as LPA, for PPAR*γ* is complicated by their poor water solubility and by the need to physically separate PPAR*γ*-bound and -free ligands for measuring the *K*_*d*_. Poor water solubility leads to a high degree of nonspecific binding and reduces physiological significance [60]. However, Davies et al. first reported an oxidatively fragmented alkyl phospholipid in oxidized LDL (oxLDL), termed azPC, as a high-affinity phospholipid-derived ligand of PPAR*γ* [54]. Radiolabeled azPC was shown to bind PPAR*γ* with an affinity of approximately 40 nM, which is equivalent to TZD drugs, like rosiglitazone [54]. Shortly after, our group identified a naturally occurring ether analog of LPA, alkyl-LPA (Figure 1), a high-affinity partial agonist of PPAR*γ* [53]. Alkyl-LPA, but not acyl-LPA, accumulates in mox-LDL and more potently activates PPAR*γ*-mediated transcription compared to acyl-LPA [53]. Binding studies using *γ*-globulin and polyethylene glycol 8000 (PEG) precipitation showed that binding of radiolabeled alky-LPA was concentration dependent and saturable with an apparent *K*_*d*_ of 60 nM [53]. To determine the molecular basis of the high-affinity binding to PPAR*γ*, we used molecular modeling techniques to computationally dock alkyl-LPA within the PPAR*γ* pocket residues [53]. Ligand-binding specificity was imposed by the size and charge of the amino acids lining the ligand-binding pocket [61]. Alkyl-LPA hydrocarbons did not form hydrogen bonds with the 2 histidines (His-323 and His-449) as rosiglitazone does [53]. In contrast, the phosphate head group of alkyl-LPA is predicted to make a salt bridge with Arg-288, a residue that is not engaged by rosiglitazone [53]. R288A mutants showed reduced alkyl-LPA binding and reduced transcriptional activity in response to 10 *μ*M alkyl-LPA [53]. The Arg-288 residue likely plays a role in distinguishing the interactions of PPAR*γ* with alky-LPA versus rosiglitazone [53]. These results highlight distinct interactions between alkyl-LPA and rosiglitazone with select residues within the PPAR*γ*-ligand-binding domain.

## 3. Synthetic and Natural PPAR*γ* Antagonists

As mentioned above, many studies have investigated the roles of PPAR*γ* agonists in many diseases, such as cardiovascular disease in diabetics [62], autoimmune encephalomyelitis [63], lung disease [64], and Alzheimer's disease [65]. However, relatively few reports have described the mechanisms of PPAR*γ* antagonists. Wright et al. reported that bisphenol A diglycidyl ether (BADGE), which is a compound used in the manufacture of industrial plastics, is a synthetic antagonist of PPAR*γ* with a *K*_*d*_ of 100 *μ*M [66]. BADGE can antagonize rosiglitazone's activation of PPAR*γ* transcriptional activity and adipogenic action in 3T3-L1 and 3T3-F442A preadipocyte cells. BADGE also affected the expression of different adipocyte-specific markers, including adipocyte fatty acid-binding protein (aP2), glycerol-3-phosphate dehydrogenase (GPD), glucose transporter type 4 (GLUT4), and adipsin. However, Bishop-Bailey et al. reported that BADGE is a PPAR*γ* agonist in a human urinary bladder carcinoma cell line, ECV304, that stably expresses the rat acyl-CoA PPAR response element (PPRE) linked to drive the expression of luciferase [67]. Furthermore, Nakamura et al. reported that BADGE is a PPAR*γ* agonist in the macrophage-like cell line, RAW 264.7, and suppressed tumor necrosis factor-alpha (TNF-*α*) production [68]. These reports suggest that the regulation of PPAR*γ* activation or inhibition may have greater cell-type specificity than previously thought. Rieusset et al. reported that dimethyl *α*-(dimethoxyphosphinyl)-*p*-chlorobenzyl phosphate (SR-202) is a selective synthetic PPAR*γ* antagonist that blocks adipocyte differentiation induced by troglitazone [69]. SR-202 attenuates agonist-induced PPAR*γ* transcriptional activity (IC_50_ = 140 *μ*M) and improves insulin sensitivity in diabetic ob/ob mice. It also increases HDL levels in rats, indicating its potential for treating obesity and type II diabetes. PD068235, a reported PPAR*γ* antagonist, inhibited rosiglitazone-dependent PPAR*γ* transcriptional activity with an IC_50_ of 0.84 *μ*M and prevented association with the agonist-induced coactivator, SRC-1 [70]. PD068235 itself did not significantly change PPAR*γ* transcriptional activity; however, cotreatment with rosiglitazone dose dependently decreased PPAR*γ* transcriptional activity.

2-chloro-5-nitrobenzanilide (GW9662) is a potent, irreversible, and selective PPAR*γ* antagonist (IC_50_ = 3.3 nM) in both cell-free and cell-based assays, which acts by covalently modifying a cysteine residue (Cys 286) in the PPAR*γ*-LBD [71]. Interestingly, GW9662 enhanced the inhibitory effect of the agonist rosiglitazone on breast cancer cells rather than rescuing tumor growth, suggesting that PPAR*γ* activation may not be involved in inhibition of survival and cell growth caused by agonists [72]. In 2002, a very potent and selective non-TZD-derived PPAR*γ* antagonist, 2-chloro-5-nitro-*N*-4-pyridinylbenza (T0070907), was newly identified [73]. It was reported to bind PPAR*γ* with a high affinity (IC_50_ = 1 nM) and block adipocyte differentiation. Furthermore, T0070907 promoted the recruitment of the transcriptional corepressor NCoR [74] as a result of binding to PPAR*γ* and causing conformational changes. In contrast, very few endogenous PPAR*γ* antagonists have been described. Prostaglandin F2*α* (PGF2*α*) was first described as naturally occurring PPAR*γ* antagonist; it potently inhibits adipocyte differentiation in 3T3-L1 cells [75]. A main step in the synthesis of PGF2*α* is the conversion of arachidonic acid into the unstable intermediate prostaglandin H2 (PGH2) through the activity of cyclooxygenase (COX) [76]. PGF2*α* induces MAP kinase activation, leading to the phosphorylation of PPAR*γ* at Ser 112. This effect suggests that PGF2*α* indirectly antagonized PPAR*γ* induction and inhibited adipocyte differentiation [75]. Our recent work identified cPA (Figure 1) as a naturally occurring PPAR*γ* antagonist generated by phospholipase D2 (PLD2). cPA is an analog of LPA with a 5-atom ring linking the phosphate to 2 of the glycerol carbons. cPA is found in diverse organisms, from slime mold to humans [77, 78]; however, its functions are largely unknown. The concentration of cPA in human serum is estimated to be ~10 nM, which is ~100-fold lower than that of LPA. Although cPA is structurally similar to LPA, it has several unique actions. cPA inhibits cell proliferation, induces actin stress fiber formation, promotes differentiation and survival of cultured embryonic hippocampal neurons, inhibits LPA-induced platelet aggregation, and suppresses cancer cell invasion and metastasis *in vitro* and *in vivo* [79–81].

## 4. Transcriptional Corepressors and Epigenetic Modifications

### 4.1. PPAR*γ* Ligands and Epigenetic Control

We showed that cPA negatively regulates PPAR*γ* functions by stabilizing the SMRT-PPAR*γ* complex [14]. Epigenetic mechanisms are often responsible for regulating specific gene activation and repression [82]. DNA methylation and histone modification serve as epigenetic markers for active or inactive chromatin. Gene repression through posttranslational modification is targeted to specific DNA sites through DNA methylation [83]. Epigenesis plays a vital role in the regulation of gene expression; DNA methylation plays an important role in these structural changes [84]. DNA methylation occurs on cytosine bases and is catalyzed by DNA methyltransferases.

 In general, DNA methylation is thought to repress gene transcription through either directly preventing the binding of transcription factors or by creating binding sites for methyl-binding proteins [85]. Several studies have reported that epigenetic regulatory mechanisms are involved in the transcriptional activation of PPAR*γ* in 3T3-L1 adipocytes [86]. Fujiki et al. recently reported that the *PPARγ* gene is regulated by DNA methylation of its promoter region, which reduces expression of PPAR*γ* [87]. These findings suggest that DNA methylation of the PPAR*γ* promoter contributes to its expression during adipocyte differentiation.

 Acetylation of core histone proteins occurs on specific lysine residues, creating a neutral charge that loosens DNA-histone interactions and permits the binding of transcription factors [88]. Many proteins have been identified as coregulators that can be recruited by nuclear receptors to affect transcriptional regulation. The corepressor for PPAR*γ* is a protein complex containing histone deacetylase 3 (HDAC3) and SMRT or NCoR. A number of PPAR*γ* interacting partners have been identified, many of which are known epigenetic regulators, including HDAC3 [89, 90]. HDACs repress gene expression by deacetylating histones and condensing chromatin. Many nuclear receptors, including PPAR*γ* in the unligated or antagonist-bound state, repress transcription by recruiting corepressors [91, 92], which bind to the heterodimer to suppress target gene activation. The nuclear receptor corepressor NCoR and SMRT are structurally related and extensively studied corepressors. NCoR and SMRT are encoded by separate loci but share a similar modular structure. The N-terminus contains several repression domains (RDs). The PPAR*γ* AF2 domain is accessible and can interact with the extended LXXXIXXXL consensus motif of NR corepressors [93]. These corepressor complexes significantly regulate the control of transcription in inactive states [8]. NCoR and SMRT nucleate a core corepressor complex that contains HDAC3, transducin *β*-like 1 (TBL1), TBL1-related protein (TBLR1), and G protein pathway suppressor 2 (GPS2), forming a functional holocomplex [94]. HDAC3 is found in a tight complex with SMRT and NCoR in diverse repression pathways [95]. These 2 corepressors recruit HDAC3 to specific promoters, where it deacetylates histones and mediates silencing of the corresponding genes. TBL1 is a 6 WD-40 repeat-containing protein (also known as beta-transducin repeat) that was identified as a subunit of the SMRT complex [96]. Both TBL1 and TBLR1 interact directly with SMRT and NCoR but not with HDAC3. They activate PPAR*γ*-dependent transcription in response to rosiglitazone. The transcriptional activity of PPAR*γ* is controlled by DNA-binding activity and nuclear receptor cofactors [97]. These corepressor complexes associate with a variety of factors that mediate transcription repression.

### 4.2. cPA-Induced Corepressor SMRT and Interaction with Human Diseases

**Figure 2:**
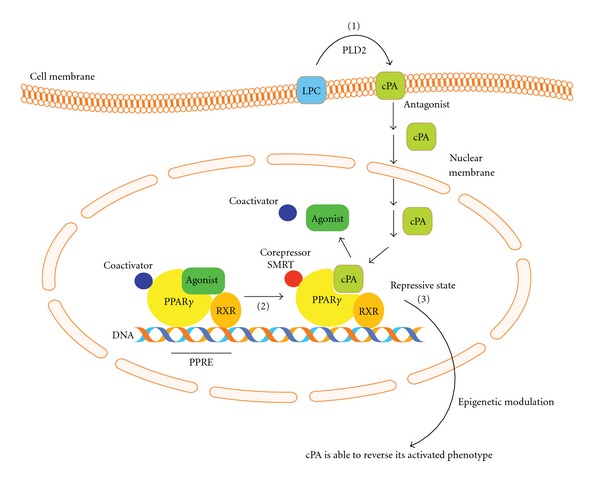
Schematic diagram of the PPAR*γ* signaling. cPA is generated intracellularly in a stimulus-coupled manner by the PLD2 enzyme (1). cPA inhibits PPAR*γ* activation and stabilizes binding of PPAR*γ* corepressor SMRT (2). Agonists (LPA, alkyl-LPA, and rosiglitazone) activate PPAR*γ* and promote downstream signals, whereas cPA negatively regulates PPAR*γ*. cPA stabilizes PPAR*γ*-SMRT corepressor complex and inhibits PPAR*γ*-mediated postsignal transduction (3).

Our recent report used a corepressor 2-hybrid assay to show that cPA negatively regulates PPAR*γ* function by stabilizing the SMRT-PPAR*γ* complex (Figure 2) and blocks rosiglitazone-stimulated adipogenesis and lipid accumulation in 3T3-L1 and RAW246.7 macrophage-like cells [14]. This ligand-dependent corepressor exchange results in transcriptional repression of genes involved in the control of insulin action as well as a diverse range of other functions [98]. We also demonstrated that activation of PLD2-mediated cPA production by insulin or topical application of cPA together with PPAR*γ* agonists prevents neointima formation, adipocytic differentiation, lipid accumulation, and upregulation of PPAR*γ* target genes [13, 14]. Atherosclerosis is the leading cause of death among cardiovascular diseases. Neointima formation is a common feature of an atherosclerotic artery and is characterized by smooth muscle cell (SMC) proliferation and extracellular matrix deposition in the vascular intimal layer. Yoshida et al. first reported that LPA and species containing unsaturated LPA (16 : 1, 18 : 1 and 18 : 2) induced neointima formation when injected into the rat carotid artery [99]. Furthermore, LPA and alkyl-LPA induced neointima formation through the activation of PPAR*γ*, whereas cPA inhibited PPAR*γ*-mediated arterial wall remodeling in a noninjury infusion model [13, 14]. These results suggest that PPAR*γ* is required for LPA-induced neointima formation. PPAR*γ* antagonists should continue to be developed, as they have the clinical potential for preventing neointimal vascular lesions.

## 5. Conclusion

In this paper, we have focused on recent developments elucidating the role of lysophospholipids in intracellular signaling and PPAR*γ* activation and inhibition. Our proposed mechanism of action for the cPA-PPAR*γ* axis is summarized in Figure 2. Lysophospholipids fulfill dual role as mediators, through the activation of cell surface GPCRs, and as intracellular second messengers, through the activation and inhibition of PPAR*γ*. PPAR*γ*-corepressor interactions are physiologically relevant, as reports have demonstrated the involvement of chromatin-modifying cofactors in diseases, such as cancer [100] and metabolic syndrome diseases [101]. However, the physiological context of these compounds in PPAR*γ* signaling is still unclear. Further clarification of the PPAR*γ*-cPA axis could allow the synthesis of novel medicines that modulate PPAR*γ*.
